# Inflammatory signature of post-COVID-19 depression

**DOI:** 10.1192/j.eurpsy.2023.325

**Published:** 2023-07-19

**Authors:** M. Palladini, M. G. Mazza, V. Aggio, S. Spadini, F. Calesella, S. Poletti, P. Rovere-Querini, F. Benedetti

**Affiliations:** 1 Psychiatry & Clinical Psychobiology Unit, Division of Neuroscience, IRCCS San Raffaele Hospital; 2 Vita-Salute San Raffaele University, Milano, Italy

## Abstract

**Introduction:**

Persisting and disabling depressive symptomatology represent a prominent feature of the post-acute COVID-19 syndrome. Sars-CoV-2-induced immune system dysregulation mainly result in a cytokine storm. Once in the brain, inflammatory mediators negatively affect neurotransmission, microglia activation, and oxidative stress, possibly disrupting critical brain neurocircuits which underpin depressive symptoms. So far, only inflammatory markers based on leukocyte counts have been linked to depressive outcome in COVID survivors. However, an accurate immune profile of post-COVID depression has yet to be elucidated.

**Objectives:**

Identify inflammatory mediators that predict post-COVID depression among a panel of cytokines, chemokines, and growth factors, with a machine learning routine.

**Methods:**

88 COVID age- and sex-matched survivors’ (age 52.01 ± 9.32) were screened for depressive symptomatology one month after the virus clearance through the Beck Depression Inventory (BDI-13), with 12.5% of the individuals scoring in the clinical range (BDI-13 ≥ 9). Immune assay was performed through Luminex system on blood sampling obtained in the same context. We entered 42 analytes into an elastic net penalized regression model predicting presence of clinical depression, applied within a 5-fold nested cross-validation machine learning routine running in MATLAB. Significance of predictors was evaluated according to variable inclusion probability (VIP), as returned by 5000 bootstraps. Socio-demographics, previous psychiatric history, hospitalization, time after discharge were used as covariates.

**Results:**

The model reached a balance accuracy of 73% and AUC of 77%, correctly identifying 73% of people suffering from clinically relevant depressive symptoms (Figure1). Depressive symptomatology was predicted by high levels of CCL17, ICAM-1, MIF, whereas CXCL13, CXCL12, CXCL10, CXCL5, CXCL2, CCL23, CCL15, CCL8, GM-CSF showed a protective effect (Figure2).

**Image:**

**Figure:**
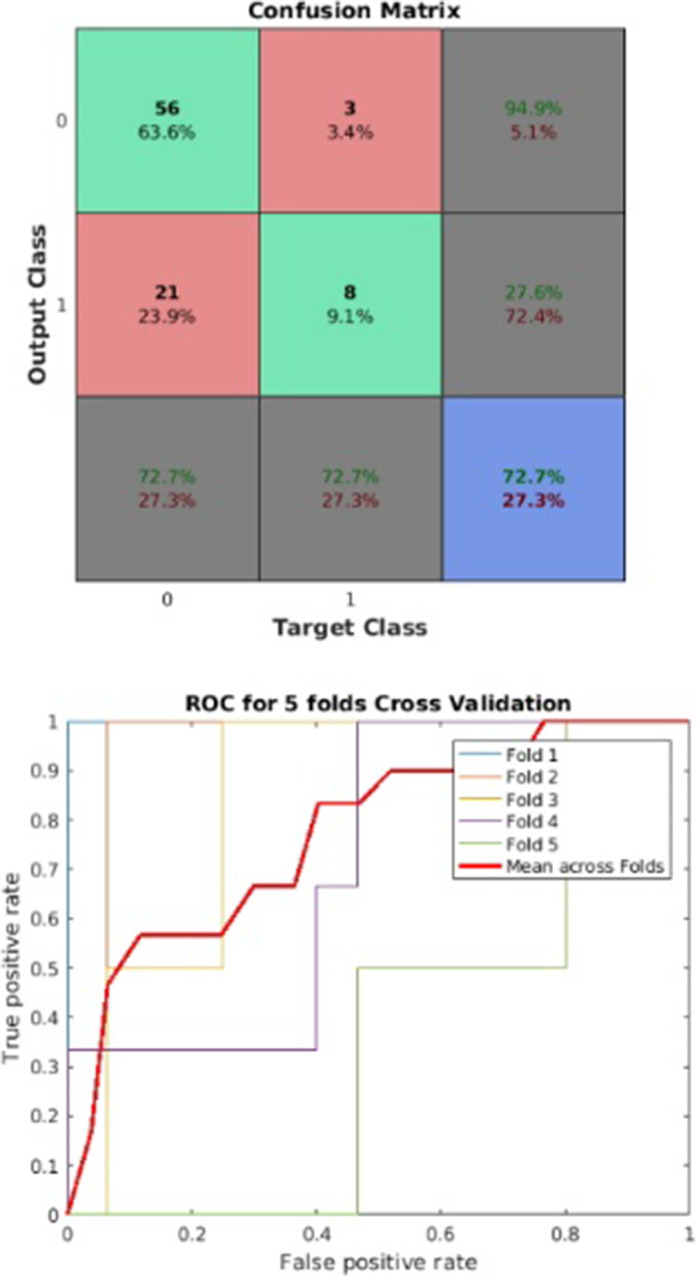

**Image 2:**

**Figure:**
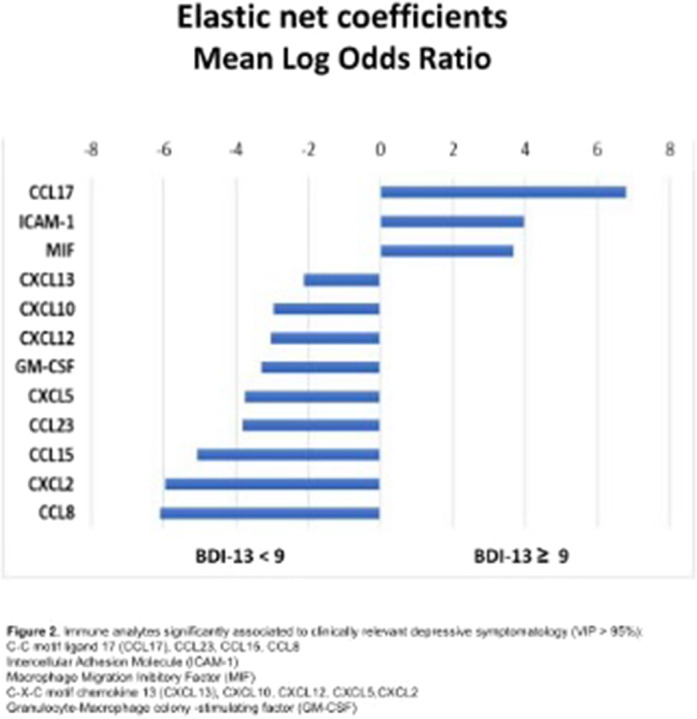

**Conclusions:**

This is the first study highlighting a putative inflammatory signature of post-COVID depression. Consistently to the immune profile of Major Depressive disorder, upregulation of innate immunity mediators seems to foster depressive symptoms in the aftermath of COVID. Interestingly, recruiters of B and T cells promoting a physiological adaptive response to viral infection also mitigate its psychiatric sequelae. Understanding the biological basis of post-COVID depression could pave the way for personalized treatments capable of reducing its add-on burden.

**Disclosure of Interest:**

None Declared

